# Supplementary material for: Machine learning climbs the Jacob’s Ladder of optoelectronic properties
Source: Nat Commun. 2025 Aug 31;16:8142. doi: 10.1038/s41467-025-63355-9 (PMC12398487; doi:10.1038/s41467-025-63355-9)
Supplement: Supplementary file 1 — Supplementary Information [file 41467_2025_63355_MOESM1_ESM.pdf]

Supplementary Information for "Machine learning climbs the Jacob's Ladder of optoelectronic properties"

Malte Grunert,[1] Max Großmann,[1] and Erich Runge[1]

[1] *Institute of Physics and Institute of Micro- and Nanotechnologies, Technische Universität Ilmenau, 98693 Ilmenau, Germany*

(Dated: August 15, 2025)

SUPPLEMENTARY NOTE 1: PENDANT OF FIG. 4 OF THE MAIN TEXT, ALBEIT EVALUATED WITH THE FULL TRAINING SET

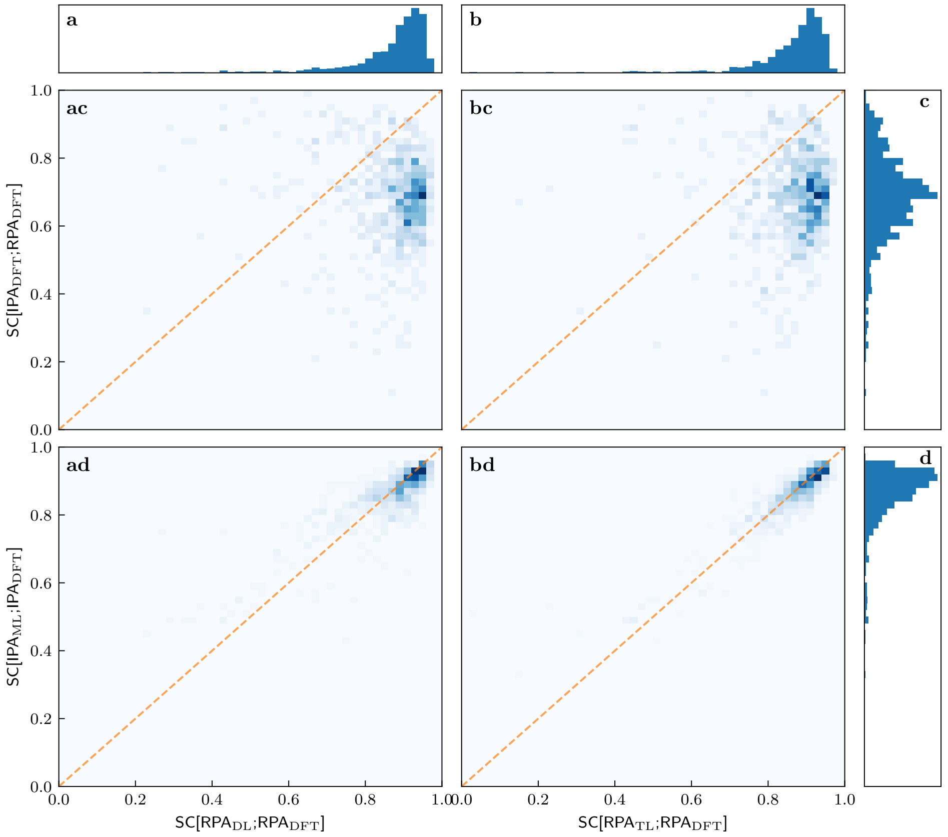

Supplementary Figure 1. **Correlations between various similarity coefficients on the test set after training on 4610 materials.** For a detailed description, we refer to Fig. 4 of the main text. As expected, there is an improvement for both strategies, with the improvement being more pronounced for the direct learning strategy. The linear relation between SC[IPA$_{ML}$;IPA$_{DFT}$] and both SC[RPA$_{DL}$;RPA$_{DFT}$] and SC[RPA$_{TL}$;RPA$_{DFT}$] is very clearly visible.

SUPPLEMENTARY NOTE 2: OPTIMIZED HYPERPARAMETERS

In Supplementary Tab. 1, we show the optimal architecture hyperparameters that were found for the direct learning strategy, whereas in Supplementary Tab. 2, we show the optimal optimizer hyperparameters that were found for both the transfer learning and direct learning strategy. We note that a large range of architecture and optimizer hyperparameters perform similarly. For convenience, Supplementary Fig. 2 summarizes schematically the OptiMate architecture.

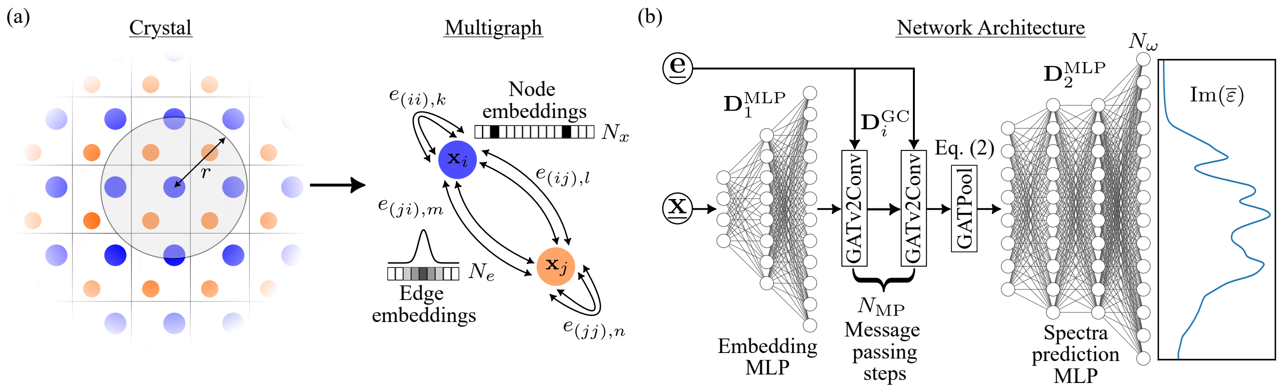

Supplementary Figure 2. **Schematic presentation of the OptiMate models and their hyperparameters.** (a) Schematic illustration of the conversion of a 2D toy crystal into its multigraph representation, chosen for clearer visualization. Connections between atoms are determined by all periodic neighbors within a fixed cutoff radius r. The nodes $\underline{\mathbf{x}}$ encode atom types by periodic table groups and periods using one-hot encoding, while bond distances are captured by Gaussian expanded edge embeddings $\underline{\mathbf{e}}$. The approach for the 3D case is analogous. (b) Architecture of the OptiMate models, highlighting the integration of encoded atomic and bond information. The dimension of the MLP layers ($\mathbf{D}_1^{\mathrm{MLP}}$, $\mathbf{D}_2^{\mathrm{MLP}}$), the number of message passing steps (N_{MP}), and the parameters of the ith GATv2Conv layers ($\mathbf{D}_i^{\mathrm{GC}}$) are all architecture parameters that were also optimized during the hyperparameter optimization described in the SM. Figure and Caption reproduced from Grunert *et al.*, Phys. Rev. Mater. **8**, L122201 (2024), DOI: 10.1103/PhysRevMaterials.8.L122201

Training-set size	Node-embedding MLP	GAT	Spectra-prediction MLP
100	96, 96, 96	[192, 2]	512, 512, 512
300	96, 96, 96	[384, 4]	512, 512
1000	96, 96, 96	[192, 8], [96, 8], [96, 2]	2048, 2048, 2048
3000	48, 48, 48	[384, 8]	2048, 2048, 2048
4610	96, 96	[192, 8], [192, 2]	2048, 2048, 2048

Supplementary Table 1. **Optimized architecture hyperparameters for the direct learning strategy.** For the node-embedding and spectra-prediction multilayer perceptrons (MLPs), the numbers indicate the number of nodes in each hidden layer. The node embedding MLP has an additional input layer of dimension 13, the spectra prediction MLP has an additional input layer with a dimension equal to the output dimension of the final GAT, and an additional output layer of dimension 2001 determining the spectral resolution. For the graph attention layers (GATs), the first number indicates the output dimension of each attention head and the second number indicates the number of attention heads. The actual output dimension of the GATs is equal to the product of the two numbers.

Strategy	Training-set size	Learning rate	L_2 Regularization	Batch size
DL	100	10^{-4}	10^{-4}	10
DL	300	10^{-5}	0	20
DL	1000	10^{-3}	10^{-4}	32
DL	3000	10^{-3}	10^{-4}	128
DL	4610	10^{-3}	10^{-6}	128
TL	100	10^{-5}	10^{-6}	32
TL	300	10^{-5}	10^{-4}	64
TL	1000	10^{-5}	10^{-4}	128
TL	3000	10^{-5}	10^{-4}	128
TL	4610	10^{-5}	10^{-4}	128

Supplementary Table 2. **Optimal optimizer hyperparameters.** Given are the optimal learning rate, the L_2 regularization coefficient, and the batch size.

		Training			Test		
Strategy	Training-set size	Median MSE	Median MAE	Median SC	Median MSE	Median MAE	Median SC
DL	100	0.043	0.138	0.89	0.393	0.403	0.716
DL	300	0.085	0.186	0.863	0.262	0.339	0.754
DL	1000	0.046	0.139	0.897	0.160	0.249	0.827
DL	3000	0.015	0.082	0.939	0.075	0.173	0.874
DL	4610	0.009	0.064	0.954	0.052	0.140	0.900
TL	100	0.014	0.083	0.934	0.133	0.243	0.834
TL	300	0.007	0.056	0.958	0.101	0.197	0.863
TL	1000	0.009	0.059	0.956	0.077	0.170	0.881
TL	3000	0.010	0.0627	0.955	0.059	0.148	0.893
TL	4610	0.018	0.087	0.937	0.061	0.151	0.893
TL	up to 2 sites	0.008	0.049	0.961	0.225	0.299	0.791
TL	up to 3 sites	0.013	0.071	0.953	0.118	0.180	0.848
TL	up to 4 sites	0.016	0.080	0.945	0.086	0.184	0.870
TL	up to 5 sites	0.012	0.069	0.95	0.079	0.176	0.879
TL	up to 6 sites	0.012	0.069	0.950	0.075	0.167	0.884

Supplementary Table 3. **Error measures of the different models**. The training set errors and SCs are evaluated on the respective training set, while the test set errors are evaluated on the full test set.

$G_{\max}$ (mRy)	CPU Time (min)	SC
0	6.8 (4.3)	0.68 (0.69)
2000	221.6 (69.3)	0.97 (0.97)

Supplementary Table 4. **Computational time and Similarity Coefficient for intermediate RPA calculations.** Shown are the mean (median) CPU times and Similarity Coefficients for $\mathrm{Im}(\bar{\varepsilon})$ for RPA calculations with non-converged $G_{\max}$-values, averaged over the entire dataset. The Similarity Coefficients are obtained by comparing to the spectra with converged $G_{\max}$-values. We note that the converged $G_{\max}$-value depends on the material and the direction over which the dielectric function is evaluated. Only $G_{\max} = 0$ mRy (i.e., IPA spectra) and $G_{\max} = 2000$ mRy are shown, as higher values of $G_{\max}$ are not present for all materials, as lower values might already be converged.

SUPPLEMENTARY NOTE 3: DISTRIBUTION OF ELEMENTS IN DATA BASE

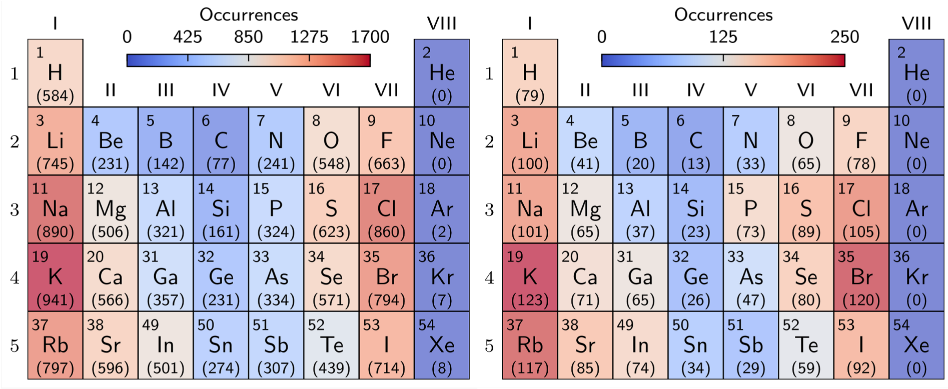

Supplementary Figure 3. **Distribution of elements in training and test data set.** Periodic table reduced to the main group elements of the first five periods, showing the distribution of the elements in the full training set (left) and the test set (right). The colors indicate the number of compounds in which an element occurs (also shown in parentheses below the respective element symbol).

SUPPLEMENTARY NOTE 4: PARITY PLOTS

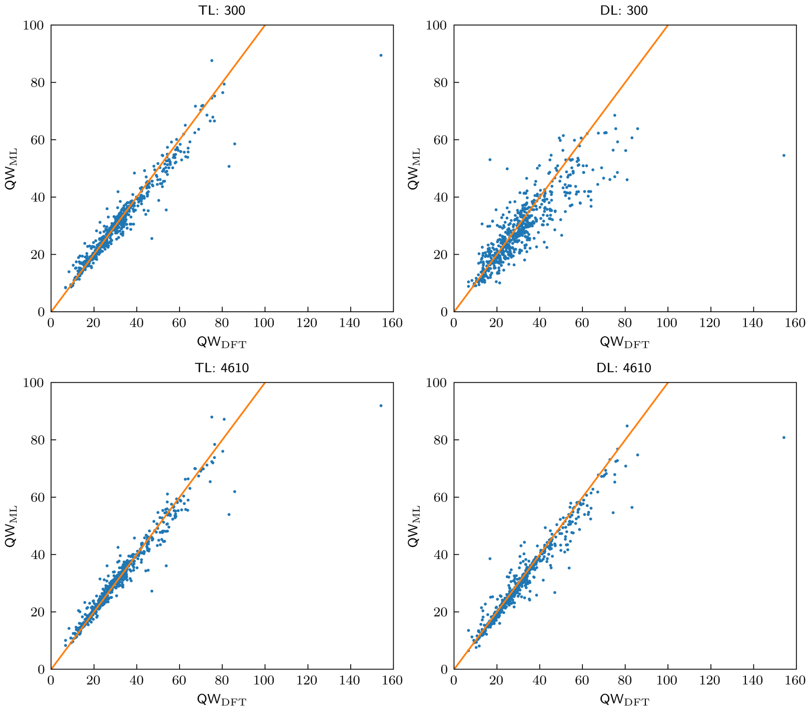

Supplementary Figure 4. **Parity plots of the approximate quantum weight.** Parity plots between the (approximate) quantum weight of the spectra for four different models, evaluated over the entire test set. The quantum weight is approximated as $\mathrm{QW} = \int \mathrm{Im}(\varepsilon(\omega))\, d\omega$ [1, 2]. It can be seen that TL already yields quite good parity with just 300 data points, but DL only performs well with a much larger training set.

SUPPLEMENTARY NOTE 5: TRAINING OF THE SC PREDICTION MODEL

We do not separately optimize the hyperparameters for the SC prediction model and instead use the architecture hyperparameters of the original OptiMate model for the prediction of $\mathrm{Im}(\bar{\varepsilon}(\omega))$ with a broadening of 300 meV [3], i.e., a node embedding MLP with parameters $[48, 48]$, GAT parameters of $[48, 4]$, $[96, 4]$, and a spectra prediction MLP with parameters $[1024, 1024, 1024]$ (see caption of Supplementary Tab. I). As mentioned in the main text, the final output size of the model is changed from 2001 to 1. For optimizer hyperparameters, we use a learning rate of 10^{-3} and no weight decay. We train for 200 epochs with a batch size of 64.

[1] M. Grunert, M. Großmann, and E. Runge, Discovery of sustainable energy materials via the machine-learned material space, Small, 2412519 (2025).

[2] N. T. Hung, R. Okabe, A. Chotrattanapituk, and M. Li, Universal ensemble-embedding graph neural network for direct prediction of optical spectra from crystal structures, Adv. Mater. **36**, 2409175 (2024).

[3] M. Grunert, M. Großmann, and E. Runge, Deep learning of spectra: Predicting the dielectric function of semiconductors, Phys. Rev. Materials **8**, L122201 (2024).
